# Supplementary material for: Characterization of a New Citrus Mutant Induced by Gamma Irradiation with a Unique Fruit Shape, Gwonje-Early, and Determination of Specific Selection Markers Using Allele-Specific PCR
Source: Plants (Basel). 2024 Mar 21;13(6):911. doi: 10.3390/plants13060911 (PMC10974580; doi:10.3390/plants13060911)
Supplement: Supplementary file 1 [file plants-13-00911-s001.zip › plants-2862026-supplementary.pdf]

Table S1. SNP detection in samples

Sample	No. of Total SNPs	No. of Homozygous SNPs[1]	No. of Heterozygous SNPs[2]	No. of Other SNPs[3]
WT	1,198,650	8,208	572,811	617,631
Gwonje-early	1,204,414	7,339	604,548	592,527

[1] In the same SNP types, 90% of sample reads mapped to the reference genome. [2] In the same SNP types, 40%–60% of sample reads mapped to the reference genome. [3] Not classified as homozygous or heterozygous

Table S2. SNP classification by genome annotation

Sample	Total SNPs	Classified SNPs[1]	IGR[2] & gene structure	Total	Homozygous	Heterozygous	Others[3]
WT	1,198,650	1,083,127	intergenic	715,536	5,577	318,289	391,670
			genic region	367,591	1,338	209,859	156,394
			exon	164,743	605	94,330	69,808
			intron	212,665	776	120,993	90,896
Gwonje-early	1,193,106	1,078,912	intergenic	719,688	4,927	334,369	380,392
			genic region	370,813	1,204	224,700	144,909
			exon	165,449	561	100,802	64,086
			intron	215,387	673	129,724	84,990

[1] Loci where genes were organized in the reference gene annotation (gff) file and could be classified as genic/intergenic. [2] Intergenic region. [3] Not classified as homozygous or heterozygous

Table S3. InDel detection in samples

Sample	No. of Total InDel Variants		No. of Homozygous Variants[1]		No. of Heterozygous Variants [2]		No. of Other Variants [3]	
	Total (InDels)	In/Del	Total (InDels)	In/Del	Total (InDels)	In/Del	Total (InDels)	In/Del
WT	172,259	88,264/83,995	3,751	1,600/2,151	57,930	29,136/28,794	110,578	57,528/53,050
Gwonje-early	167,284	84502/82782	4,050	1693/2,357	55,773	27,804/27,969	107,461	55,005/52,456

[1] In the same SNP type, 90% of sample reads mapped to the reference genome. [2] In the same SNP type, 40%~60% of sample reads mapped to the reference genome. [3] Not classified as homozygous or heterozygous

Table S4. InDel classification by genome annotation

Sample	Total InDels	Classified InDel variants[1]	IGR[2] & gene structure	Total	Homozygous	Heterozygous	Other[3]
WT	172,259	155,928	intergenic	110,566	2,429	35,781	72,187
			genic region	45,362	632	17,495	27,145
			exon	11,128	295	4,149	6,684
			intron	35,441	359	13,863	21,219
Gwonje-early	172,154	155,751	intergenic	106,902	2,621	34,280	70,001
			genic region	44,352	661	17,075	26,616
			exon	10,930	297	3,957	6,676
			intron	34,601	390	13,610	20,601

[1] Loci where genes were organized in the reference gene annotation (gff) file and could be classified as genic/intergenic. [2] Intergenic region. [3] Not classified as homozygous or heterozygous

※ In cases where the reference gene position overlaps or due to the anti-sense form of the gene, the number of exons and introns may be counted twice.

Table S5. Homo-type SNPs and InDels annotated in gene regions

Type	WT	*Gwonje-early*	Locus ID	Protein ID	Description	Species
SNP	A	T	CUMW_094980	XP_006486565.1	Glutamate receptor 3.2	*Citrus sinensis*
SNP	G	T	CUMW_259270	GAY67788.1	Hypothetical protein CUMW_259270	*Citrus unshiu*
InDel	*	–1A	CUMW_151040	XP_006421650.1	NO-associated protein 1	*Citrus clementina*

Table S6. Primers used in AS-PCR

	Primer Name		Primer Sequence	
1	GJ-SNP1 F	5-	CAACAGGAAAGCAGGCCAATG	-3
	GJ-SNP1 R	5-	TGCTGAGCCAAGTGGTTACA	-3
	Con-SNP1 R	5-	GCTGCACCCCGGATTTTCTT	-3
2	GJ-SNP2 F	5-	GTCGTTGCAAGAACCCTGT	-3
	GJ-SNP2 R	5-	TCATTGAGAAGATTATCACTACTGT	-3
3	GJ-SNP3 F	5-	GTCCTTGTGAAAAATGTTGAAGT	-3
	GJ-SNP3 R	5-	TTTCGCATGTGAAGTGTCTT	-3
4	Con-SNP4 F	5-	ATCCACCAGACATTAAGCTTCGT	-3
	GJ-SNP4 F	5-	CAGGTTGTCGCTTTTGTGAA	-3
	GJ-SNP4 R	5-	CTATTCATAACGTTTTGAGTCATAACGA	-3
5	GJ-SNP5 F	5-	GACGGTCATGATTTAATTGTTGGT	-3
	GJ-SNP5 R	5-	TTTGTCGACCGACTCATTTG	-3
